# Wharton’s jelly mesenchymal stem cells promote wound healing and tissue regeneration

**DOI:** 10.1186/scrt451

**Published:** 2014-05-02

**Authors:** Lars-Peter Kamolz, Maike Keck, Cornelia Kasper

**Affiliations:** 1 Division of Plastic, Aesthetic and Reconstructive Surgery, Research Unit for Tissue Regeneratjion, Repair and Reconstruction, Department of Surgery, Medical University of Graz, Auenbruggerplatz 29, Graz A-8036, Austria; 2 Division of Plastic and Reconstructive Surgery, Department of Surgery, Medical University of Vienna, Waehringer Guertel 18-20 A-1090, Vienna, Austria; 3 Department of Biotechnology, University of Natural Resources and Life Sciences, Muthgasse 18, Vienna A-1190, Austria

## Abstract

Wound healing requires an orchestrated integration of complex biological and molecular events, which include inflammation, proliferation and remodeling. Wharton’s jelly mesenchymal stem cells seem to promote wound healing and tissue repair. Wharton’s jelly stem cells promote fibroblast proliferation and migration, accelerate re-epithelialization and promote overall wound repair by pcrine signaling. Wharton’s jelly is an advantageous mesenchymal stem cell source because the harvest of this type of stem cells is not painful or invasive and because, beside their effect on wound healing, they seem to have a significant impact on the treatment of keloids. Furthermore, they led to better nerve regeneration, better neuroprotection and less inflammation.

## 

Wound healing requires an orchestrated integration of complex biological and molecular events, which include inflammation, proliferation and remodeling [1]. Despite the current use and availability of a wide array of wound dressings, ointments and devices [2], wound healing still remains a clinical challenge, especially in older patients, diabetic patients, heavy smokers or burned patients [1]. Such wounds, if not treated effectively, eventually end up in amputations or disfiguring scars termed hypertrophic scars and keloids, because surgical procedures such as local or free flaps go along with limited donor site availability and require a stable general health condition of the patient. There is therefore a need for new strategies to promote or at least coadjuvantly help wound healing and tissue repair.

Skin cell renewal is under the control of mesenchymal stem cells (MSCs). Skin MSCs populate the normal skin niche, remain quiescent and become active after injury, aiding in wound closure [3]. MSC pcrine signaling is suggested to be the main underlying mechanism for the enhanced wound repair effects [4]. Treatment of wounds has therefore been attempted with the application of exogenous bone marrow MSCs. Bone marrow MSCs have been reported to promote wound healing by modulating the inflammatory environment, promoting angiogenesis and vascularization, encouraging the migration of keratinocytes and inhibiting apoptosis of wound healing cells, but their isolation requires an invasive and artificial method [1].

Adipose-derived stem cells (ADSCs) are located between mature adipocytes in the adipose tissue. ADSCs are able to differentiate into a variety of different tissues such as bone, adipose tissue and cartilage both *in vitro* and *in vivo*. Preclinical and clinical studies have shown a positive effect of ADSCs on wound healing and have verified them as a suitable cell type for skin substitution [5,6].

ADSCs can relatively easily be harvested through liposuction or during abdominoplasty, but this still requires a surgical procedure. Researchers thus focused on a more advantageous MSC source, such as umbilical cord-derived Wharton’s jelly [7]. The harvest of this type of stem cells is not painful or invasive; the cells are isolated naturally with no extra surgeries and are dissected from discarded umbilical cords after birth. Isolation of ADSCs requires enzymatic digestion using collagenase, whereas Wharton’s jelly stem cells can optimally be generated by explant culture.

Moreover, human umbilical cord Wharton’s jelly mesenchymal stem cells (WJ-MSCs) have been shown to have better clinical utility. WJ-MSCs have unique properties between embryonic and adult stem cells. These cells have low-level expression of embryonic stem cell markers and satisfy the criteria recommended by the International Society of Cytotherapy for MSCs. Stem cell quality, quantity and safety can be optimized when cultured under controlled physiolocal conditions, including appropriate oxygen and nutrition supply as well as waste elimination. These can be realized in bioreactors using dynamic culturing strategies.

WJ-MSCs represent a very efficient stem cell source with reported immunoprivileged, anticancer and antifibrotic characteristics under *in vitro* conditions and in animal models [7]. Due to their reported immunoprivileged properties and universal and ever-lasting availability, their allotransplantation as an off-the-shelf therapy may represent an appropriate treatment strategy in the already compromised patients who suffer recalcitrant cutaneous wounds [3].

Indeed, caprine WJ-MSCs have already been shown to promote wound repair with minimal scarring, and the results of a recent study by Arno and colleagues suggest that WJ-MSCs enhance wound healing and tissue repair [1]. WJ-MSCs promoted fibroblast proliferation and migration, accelerated the re-epithelialization rate and promoted overall wound repair by pcrine signaling. Under culture conditions, human WJ-MSCs enhanced the expression of wound-healing promoting genes, including re-epithelialization, neovascularization and fibroproliferation. Under culture conditions, significantly enhanced proliferation and migration were observed. This is an important observation because cellular dynamics and cell migration constitute an essential step during cutaneous healing. WJ-MSCs accelerated wound closure both *in vitro* and in an *in vivo* mouse wound healing model, suggesting that human WJ-MSCs may promote wound repair. Indeed, pilot clinical studies have so far indicated that MSCs in general are safe *in vivo*, and they currently represent the most widely used stem cells in the clinical setting [8]. Accordingly, in the particular case of WJ-MSCs, 15 diabetic patients received WJ-MSCs systemically with no documented relevant safety concerns [9].

In conclusion, Wharton’s jelly is an advantageous MSC source, because the harvest of this type of stem cells is not painful or invasive and because, in addition to their effect on wound healing, they seem to have a significant impact on the treatment of keloids. Furthermore, the cells led to better nerve regeneration [10], better neuroprotection and less inflammation. Also, the isolation of WJ-MSCs seems to be more efficient and the cells have a higher capacity of proliferation and are less senescent than ADSCs. Even if proper studies comparing the wound-healing abilities of different MSC sources are still lacking in the literature, at least a new preclinical and clinical research field investigating the potential of WJ-MSCs in wounds and wound healing has arrived.

## Abbreviations

ADSC: Adipose-derived stem cell; MSC: Mesenchymal stem cell; WJ-MSC: Wharton’s jelly mesenchymal stem cell.

## Competing interests

The authors declare that they have no competing interests.
